# Idiopathische Chondrolyse beider Hüftgelenke – Fallbericht bei einer adoleszenten Patientin

**DOI:** 10.1007/s00132-020-03885-w

**Published:** 2020-02-12

**Authors:** Eckehard Schumann, Fabian Bastian Kübler, Christian Roth, Christoph‑E. Heyde, Andreas Roth

**Affiliations:** 1 Klinik und Poliklinik für Orthopädie, Unfallchirurgie und Plastische Chirurgie, Universitätsklinikum Leipzig, Leipzig, Deutschland; 2 Abteilung für Pädiatrische Radiologie, Universitätsklinikum Leipzig, Leipzig, Deutschland

**Keywords:** Entzündungshemmer, Knorpelerkrankung, Konservative Therapie, Coxa magna, Schmerzen, Antiinflammatory agents, Cartilage diseases, Conservative treatment, Coxa magna, Pain

## Abstract

Am Fallbeispiel eines 12-jährigen Mädchens wird die Diagnose und Therapie einer idiopathischen Chondrolyse der Hüftgelenke beschrieben. Die Patientin stellte sich mit intermittierenden Schmerzen und hochgradigen Funktionsstörungen beider Hüften vor. Nach klinischer Untersuchung und Beckenübersichtsröntgenaufnahme zeigte die MRT die typischen Veränderungen einer idiopathischen Chondrolyse. Die rein konservative Therapie mit einer konsequenten physiotherapeutischen Behandlung und regelmäßigen Einnahme eines nichtsteroidalen Antirheumatikums hat ein gutes klinisches Ergebnis erbracht.

## Abstract (en)

The diagnosis and treatment of idiopathic chondrolysis of the hips are described in the case of a 12-year-old girl. The patient presented with intermittent pain and extreme dysfunction of both hips. After clinical examination and pelvic X‑ray, MRI showed the typical changes of idiopathic chondrolysis. Purely conservative treatment with systematic physiotherapy and regular application of a non-steroidal anti-inflammatory drug produced a good clinical result.

## Anamnese

Wir berichten über ein 12-jähriges Mädchen, das aufgrund einer hochgradigen Funktionsstörung beider Hüften behandelt wurde. Die Erstvorstellung erfolgte aufgrund intermittierender Schmerzen, die mit einer bedarfsgerechten Einnahme eines nichtsteroidalen Antiphlogistikums behandelt wurden. Eine ambulant durchgeführte Physiotherapie vor der Diagnosestellung hatte keine relevante Beschwerdelinderung erbracht. Ein Trauma oder eine zurückliegende Infektion waren nicht erinnerlich. Die Anamnese und die Vorbefunde ergaben keine relevanten Begleit- oder Grunderkrankungen, die Familienanamnese bzgl. chronischer Gelenkerkrankungen war negativ. Aufgrund der Funktionsstörung lag eine Schulsportbefreiung vor. Orthopädische Hilfsmittel wurden nicht genutzt.

## Klinischer Befund

Klinisch zeigte sich bei der Erstvorstellung ein eutrophes Mädchen (Größe: 148 cm, Gewicht:38 kg) mit einem flüssigen Gangbild und geraden Beinachsen. Die Wirbelsäule war lotrecht bei sichtbarer Haltungsschwäche. Es zeigten sich ein vorgezogenes Kopf- und Schulterlot sowie eine aktiv korrigierbare vermehrte Kyphose der Brustwirbelsäule. Es bestanden kein Rippenberg oder Lendenwulst, keine Taillenasymmetrie sowie ein Schulter- und Beckengeradstand. Die Hüftgelenke waren ohne Druck‑, Bewegungs- oder Stauchungsschmerz. Es fand sich jedoch eine hochgradige Bewegungseinschränkung, in Extension/Flexion: 0‑0-90° bds., Abduktion/Adduktion: 30-0-30° bds., Außenrotation/Innenrotation: 20-10-0° bds. Das Drehmann-Zeichen war positiv. Die Kniegelenke und Füße stellten sich klinisch unauffällig und frei beweglich dar.

**Figure 1:**
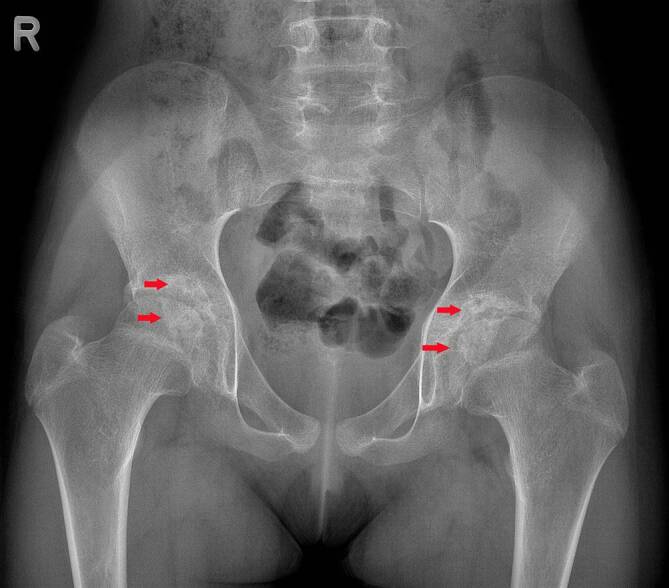

Die Beckenübersichtsröntgenaufnahme zeigte Zeichen der fortgeschrittenen sekundären Arthrose mit einer Deformierung beider Hüftköpfe und Beteiligung des Azetabulums. Weiterhin waren zystische Destruktionen sowie eine Gelenkspaltverschmälerung erkennbar (Abb. 1).

**Figure 2:**
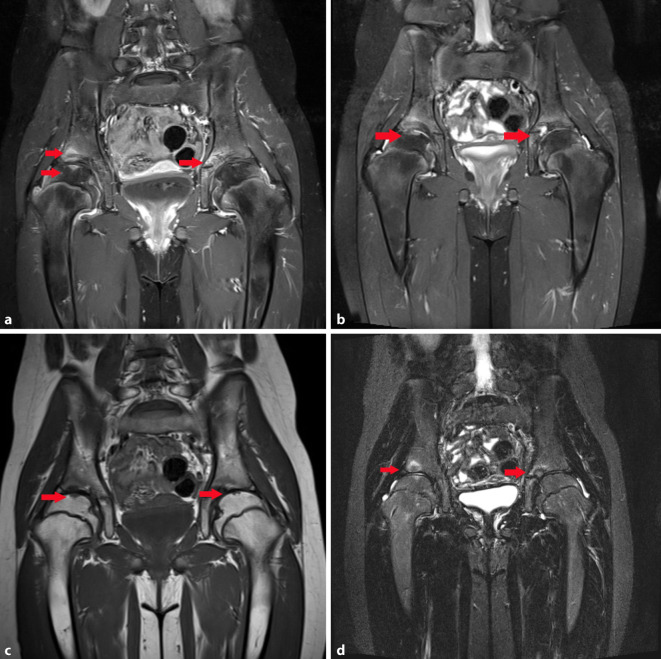

In der MRT stellten sich die in der Literatur beschriebenen typischen Veränderungen für das Vorliegen einer idiopathischen Chondrolyse in der T1- und TIRM-Wichtung dar (keilförmige Signalveränderungen hypointens in der T1-Wichtung und hyperintens in der TIRM-Wichtung). Die Veränderungen waren nicht typisch im Sinne eines Morbus Perthes. Es zeigten sich bereits eine deutliche Verschmälerung des Gelenkspaltes und des Gelenkknorpels (Abb. 2a–d; [2]).

Die weiterführende Diagnostik mittels Labor konnte eine Infektion, eine rheumatologische Erkrankung oder anderweitige strukturelle oder systemische Ursache ausschließen.

## Diagnose

Nach Auswertung aller Befunde lag eine idiopathische Chondrolyse der Hüftgelenke bds. vor. Die Bildgebung zeigte die typischen, in der Literatur beschriebenen Veränderungen der Hüftgelenke. Die Differenzialdiagnosen konnten laborchemisch oder bildgebend ausgeschlossen werden.

## Therapie und Verlauf

**Figure 3:**
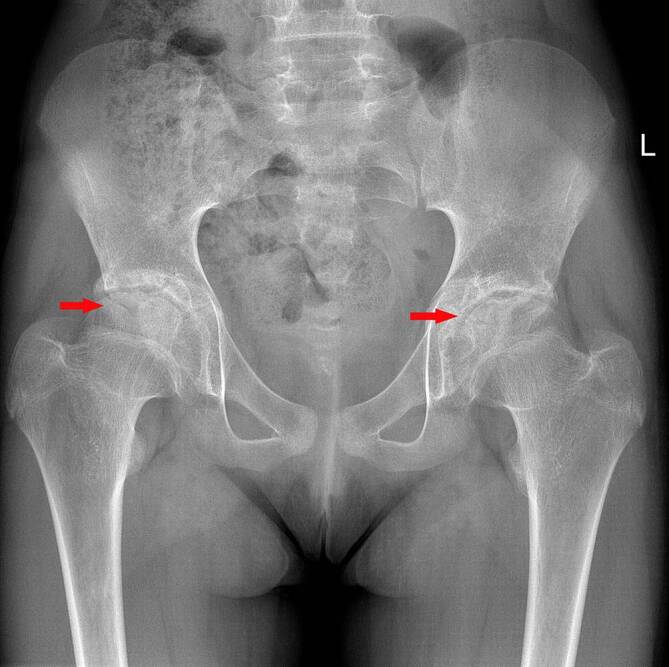

Wir leiteten eine konsequente funktionell-symptomatische Therapie mit einer kontinuierlichen physikalischen Therapie zur Funktionsverbesserung sowie eine gewichtsadaptierte regelmäßige Analgetikatherapie mit einem nichtsteroidalen Antiphlogistikum ein. Die nativradiologische Verlaufskontrolle stellte eine zunehmende Defektbildung der Epiphysen sowie eine sich entwickelnde Coxa magna dar (Abb. 3). Aufgrund der nur moderaten Schmerzsymptomatik und der im Alltag nur geringen Beeinträchtigung durch die bestandene Funktionsstörung empfahlen wir keine operative Therapie trotz der bereits fortgeschrittenen Destruktion der Gelenke. Die chirurgische Option bestünde entsprechend der Literaturempfehlung in Releaseoperationen der Muskeln und Sehnen sowie Kapsulotomien bzw. Kapselresektionen [2].

**Figure 4:**
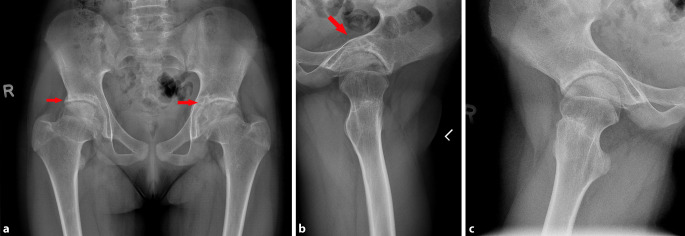

Im Verlauf von 3 Jahren berichtete die Patientin über eine kontinuierliche Beschwerderegredienz, lediglich nach längerer Belastung würde sie subjektiv ein Hinken bemerken. Eine Teilsportbefreiung in der Schule wurde beachtet. Das Fahrradfahren und Schwimmen waren problemlos möglich. Die Flexionsfähigkeit verbesserte sich bis 110°. Die Rotationsfähigkeit war unverändert. Es bestand seitens der Familie kein Wunsch bezüglich einer Intensivierung der Therapie, z. B. durch eine ergänzende Botox-Behandlung oder eine operative Therapie. Die Röntgenbildgebung sowie MRT-Verlaufskontrolle zeigte im Verlauf von 3 Jahren eine Regredienz der Defektbildung in den Femurepiphysen und azetabulär bei persistierender Coxa magna und Gelenkspaltverschmälerung (Abb. 4a–c und 5a–c).

**Figure 5:**
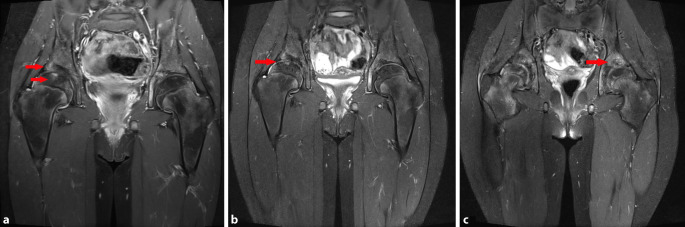

## Diskussion

Die idiopathische Chondrolyse (ICH) als eigenständiges Krankheitsbild wurde erstmals 1971 durch Jones beschrieben [10]. Der erste Bericht über die Chondrolyse des Hüftgelenkes erfolgte bereits 1930 durch Waldenström, wobei hier spezifische Ursachen, wie z. B. die Epiphyseolysis capitis femoris, Traumata oder Infektionen, angeschuldet wurden [6, 7]. Bisher wurden in der wissenschaftlichen Literatur weniger als 130 Fälle von ICH berichtet [6]. Eine gesicherte Ätiologie wurde bisher nicht beschrieben. Eine immunologische Ursache der Erkrankung wird diskutiert [2]. Die möglichen Differenzialdiagnosen, wie der Morbus Perthes, pubertäre Hüftsteife, infektiöse Arthritiden, die juvenile idiopathische Arthritis, die Epiphyseolysis capitis femoris, posttraumatische Veränderungen und längere Immobilisation als Ursache der Chondrolyse müssen ausgeschlossen werden [1, 3, 9].

Die Prävalenz ist nicht bekannt, es handelt sich bei den meisten beschriebenen Patienten jedoch um Jugendliche. Die häufigsten Beschreibungen berichten von einer unilateralen Manifestation der Erkrankung [6, 7]. Vereinzelte Krankheitsfälle im Erwachsenenalter wurden ebenfalls beschrieben [3, 7]. Laor et al. sowie Kyoung-Ho Moon et al. stellten jeweils einen Fall einer bilateralen Manifestation der Erkrankung dar [2, 7]. Das weibliche Geschlecht ist deutlich häufiger mit einem Verhältnis von 6:1 betroffen [9]. Das Krankheitsbild tritt häufiger bei dunkelhäutigen und asiatischen Patienten in der zweiten Lebensdekade auf [9, 10].

Radiologisch werden spezifische Veränderungen im Röntgen und in der MRT beschrieben. Nativradiologisch können z. B. eine periartikuläre Osteoporose, eine Gelenkspaltverschmälerung, eine Deformierung des Hüftkopfes, subchondrale Zysten oder eine Protrusio acetabuli beobachtet werden [2, 8]. In der MRT zeigen sich typischerweise ein fokaler Knorpelverlust, Gelenkerguss, Muskelatrophie, Knochenerosionen, femorale und azetabuläre Deformitäten, eine Protrusio acetabuli und ein Knochenmarksödem. Synoviale Anreicherungen sind typischerweise nicht erkennbar [3, 8]. Laor et al. berichteten jedoch über eine milde bis mäßige synoviale Mitreaktion bei den von ihnen untersuchten Patienten [2]. Das Fehlen einer synovialen Mitreaktion kann die differenzialdiagnostische Abgrenzung zur juvenilen idiopathischen Arthritis oder zur infektiösen Arthritis erleichtern [1].

Histologische Untersuchungen von betroffen Hüftgelenken zeigten eine hyperplastische Synovia mit chronischer unspezifischer Inflammation und perivaskulärer Infiltration von Lymphozyten und Plasmazellen in 95 % der Fälle [6].

Amarnath et al. haben nach Auswertung von 14 Patienten mit idiopathischer Chondrolyse eine Stadieneinteilung der Erkrankung vorgeschlagen ([9]; Tab. 1).

**Table 1:** 

Stadium	Radiologische Veränderungen
Stadium 0	Keine Auffälligkeiten
Stadium 1	Mögliche Gelenkspaltverschmälerung Keilförmige Areale mit veränderter Signalintensität in der MRT, fokale T2-Hyperintensität oder T1-Hypointensität in der proximalen Femurepiphyse (charakteristisches und frühstes MRT-Zeichen) lokalisiert im mittleren ersten Drittel des Femurkopfes in der Koronaransicht sowie synoviale Hypertrophie und Gelenkerguss
Stadium 2	Superomediales azetabuläres Ödem entlang des Knorpels an der betroffenen Hüfte, Gelenkspaltverschmälerung und keilförmige Hyperintensität im mittleren ersten Drittel des Femurkopfes in der T2-Wichtung, synoviale Hypertrophie und Gelenkerguss, häufig Zeichen der Protrusio acetabuli
Stadium 3	Ausweitung der T2-Hyperintensität in der proximalen Femurepiphyse, Kollaps des Femurkopfes, ausgeprägte azetabuläre Beteiligung, osteoporotische Veränderungen und degenerative Veränderungen mit Verlust des Gelenkspaltes (fibröse Ankylose), Vergrößerung des Hüftkopfes am Schenkelhalskopfübergang und Osteophyten

In Abhängigkeit vom Erkrankungsstadium wurde ein unterschiedliches Outcome festgestellt. So zeigten in dieser Studie die Patienten im Stadium 1 ein gutes Ergebnis im Follow up mit 7 vollständig nachweisbaren Ausheilungen bei Abwesenheit eines Ödems in den Verlaufskontrollen. Die Patienten in den Stadien 2 und 3 zeigten ein schlechtes Outcome trotz durchgeführter konservativer und operativer Therapie. Die Autoren kommen zu dem Schluss, dass die operative Therapie bei bereits fortgeschrittenem Stadium der Erkrankung kritisch bewertet werden muss [9]. Im vorliegenden Fall handelt es sich demzufolge um ein Stadium 1, übergehend zum Stadium 2, was sich sowohl in der Bildgebung, als auch im Verlauf zeigte.

Es werden in der Literatur verschiedene therapeutische Optionen zur Behandlung der idiopathischen Chondrolyse diskutiert. Die konservativen Therapien mit Physiotherapie, Einnahme von nichtsteroidalen Antirheumatika sowie Traktionsbehandlungen werden vielfach beschrieben. Eine Entlastung bei einseitiger Erkrankung ist ebenfalls erwähnt. Operative Therapie sind Releaseoperationen im Bereich der Muskeln und Sehnen sowie Kapsulotomien [2, 9].

Ein Fallbericht von einer Behandlung mit Etanercept über 1 Jahr zeigte ein gutes Resultat bezüglich Hüftgelenksfunktion und Schmerzregredienz [4]. Ein Fallbericht von 2 behandelten Patienten berichtet nach einer Botulinumtoxin-A-Behandlung, kombiniert mit einer begleitenden Physiotherapie, über gutes Outcome bezüglich Beweglichkeit und Beschwerden sowie eine Regredienz der radiologischen Veränderungen. Dabei erfolgte die ein- bzw. zweimalige Injektion im Bereich der Hüftbeuger und der Adduktorenmuskulatur mit einer nach neuropädiatrischer Empfehlung festgelegten Dosierung [5].

Die rein konservative Therapie mit einer konsequenten physiotherapeutischen Behandlung und regelmäßigen Einnahme eines nichtsteroidalen Antirheumatikums hat in dem von uns beschriebenen Fall ein gutes klinisches Ergebnis erbracht.

Bei fortgeschrittenen Erkrankungsverläufen oder älteren Patienten ist die Implantation einer Hüfttotalendoprothese notwendig, da die schmerzhafte Funktionsstörung anders nicht mehr zu behandeln ist. Dies muss jedoch insbesondere bei Patienten im Wachstumsalter kritisch indiziert werden [6, 7]. Für unsere Patientin stand diese Form der Behandlung bisher nicht zur Disposition.

Zusammenfassend ist die Therapieempfehlung aufgrund der kleinen beschriebenen Fallserien und häufigen Einzelfallberichten nicht einheitlich, eine kausale Behandlung ist bisher noch nicht bekannt. In frühen Stadien ist, wie auch der vorliegende Fall zeigt, die konservative Therapie das Mittel der Wahl.

## Fazit für die Praxis

- Bei kindlichen Hüftschmerzen sollte nach Ausschluss einer spezifischen Ursache an die seltene idiopathische Chondrolyse gedacht werden.
- Die Diagnose kann in der MRT aufgrund von spezifischen Veränderungen in den T1- und T2-Wichtungen gestellt werden.
- Eine sichere Genese der Erkrankung ist nicht bekannt, eine immunologische Ursache wird diskutiert.
- Die Therapie orientiert sich an den Beschwerden und der Funktionseinschränkung, eine kausale Behandlung ist bisher nicht bekannt.

## Abkürzungen

ICH: Idiopathische Chondrolyse
TIRM: Turbo-Inversion Recovery-Magnitude
