# Erratum zu: Idiopathische Chondrolyse beider Hüftgelenke – Fallbericht bei einer adoleszenten Patientin

**DOI:** 10.1007/s00132-021-04169-7

**Published:** 2021-09-30

**Authors:** Eckehard Schumann, Fabian Bastian Kübler, Christian Roth, Christoph‑E. Heyde, Andreas Roth

**Affiliations:** 1 Klinik und Poliklinik für Orthopädie, Unfallchirurgie und Plastische Chirurgie, Universitätsklinikum Leipzig, Leipzig, Deutschland; 2 Abteilung für Pädiatrische Radiologie, Universitätsklinikum Leipzig, Leipzig, Deutschland

**Erratum zu:**

**Orthopäde 2020**

10.1007/s00132-020-03885-w

Der Artikel *Idiopathische Chondrolyse beider Hüftgelenke – Fallbericht bei einer adoleszenten Patientin *von Eckehard Schumann, Fabian Bastian Kübler, Christian Roth, Christoph‑E. Heyde, Andreas Roth wurde ursprünglich am 12. Februar 2020 ohne „Open Access“ online auf der Internetplattform des Verlags publiziert. Die Autoren haben sich jedoch nachträglich für eine „Open Access“-Veröffentlichung entschieden. Das Urheberrecht des Artikels wurde deshalb 29. Juni 2021 in ©The Author(s) 2020 geändert.

Der Artikel wird nun unter der Creative Commons Namensnennung 4.0 International Lizenz veröffentlicht, welche die Nutzung, Vervielfältigung, Bearbeitung, Verbreitung und Wiedergabe in jeglichem Medium und Format erlaubt, sofern Sie den/die ursprünglichen Autor(en) und die Quelle ordnungsgemäß nennen, einen Link zur Creative Commons Lizenz beifügen und angeben, ob Änderungen vorgenommen wurden.

